# Fat composition in milk replacers modulates plasma cholesterol of dairy calves

**DOI:** 10.3168/jdsc.2024-0628

**Published:** 2024-10-11

**Authors:** G.B.C. Leite, J.N. Wilms, I.R.R. Castro, M.I. Marcondes, L.N. Leal

**Affiliations:** 1 Department of Animal Sciences, Washington State University, Pullman, WA 99164; 2 Trouw Nutrition Research and Development, PO Box 299, 3800 AG, Amersfoort, the Netherlands; 3 Department of Animal Sciences, Universidade Federal de Viçosa, Viçosa, MG, Brazil 36570-900

## Abstract

This study evaluated the effect of feeding milk replacers (MR) differing in fat composition on plasma cholesterol of dairy calves. Forty-five individually housed male Holstein calves (2.3 ± 0.82 d; mean ± SD) were blocked by arrival day and age. Within each block, calves were randomly assigned to (1) an MR containing a fat blend with 65% rapeseed and 35% coconut fats (RC), (2) an MR containing 65% palm and 35% coconut fats (PC), and (3) an MR containing 65% lard and 35% dairy cream (LD). All MR were isoenergetic with 30% fat, 25% protein, and 36% lactose (DM). Calves were fed 6.0 L/d from d 1 to 5, 7.0 L/d from d 6 to 9, and 8.0 L/d from d 10 to 35 at 13.5% solids. Water and chopped wheat straw were available ad libitum from arrival onward. Milk, water, and straw intakes were recorded daily. Blood was sampled and BW was measured weekly at 1300 h. Fecal scores were recorded daily after the morning meal. Growth and intakes did not differ among treatments. Plasma total cholesterol, high-density lipoprotein cholesterol, and low-density lipoprotein cholesterol concentrations were higher in calves fed RC than PC and LD, and higher in calves fed PC than LD. Additionally, plasma triglyceride was greater in calves fed PC than LD at wk 2 after arrival, while calves fed RC did not differ from other groups. The proportion of abnormal fecal scores was lower in calves fed RC than PC and LD in wk 2. The fat composition in MR significantly influenced lipid metabolism in calves but had no impact on growth.

## Highlights

• Vegetable fats from rapeseed and palm in MR raised plasma cholesterol.
• High polyunsaturated fatty acids in MR may elevate plasma cholesterol.
• C16:0 positioning in palm and lard may modulate plasma cholesterol.
• The fat blend with rapeseed and coconut improved fecal consistency.

## Graphical Abstract

**Figure:**
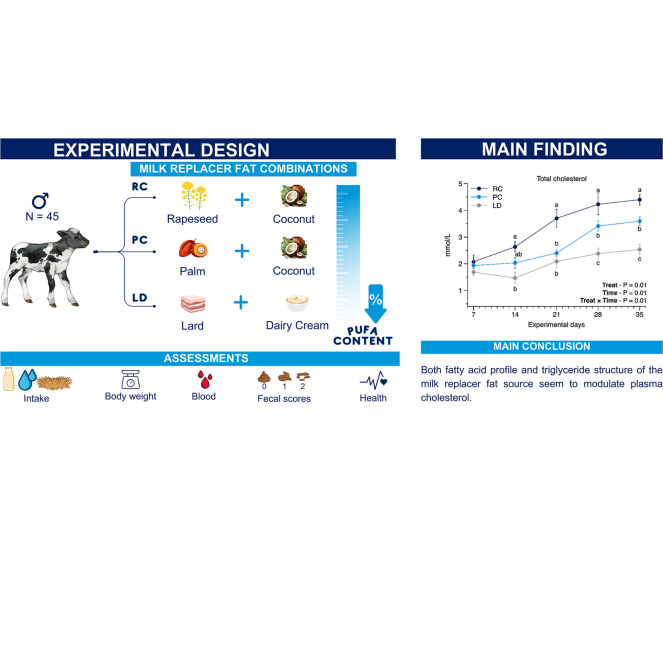
**Summary:** Formulating milk replacer (MR) for calves using alternative fat sources to milk fat leads to markedly different fatty acid profiles and triglyceride structures. Differences in fat composition can have profound effects on the lipid metabolism of calves. To further understand how fat composition affects blood cholesterol concentrations in dairy calves, 3 MR high in fat were formulated using (1) rapeseed and coconut fats (RC), (2) palm and coconut fat (PC), and (3) lard and dairy cream (LD). Plasma cholesterol was greater in calves fed MR containing vegetable fat blends than in calves fed MR containing only animal fats. In addition, calves fed MR with rapeseed and coconut had lower abnormal fecal scores compared with calves fed MR with lard and dairy cream.

Due to the high commercial value of milk fat, milk replacers (**MR**) for calves contain alternative fats derived from animal (e.g., lard, tallow) or vegetable (e.g., rapeseed, palm, coconut) sources (Huuskonen et al., 2005; Hill et al., 2007, Hocquette and Bauchart, 1999). Thus, when compared with whole milk (**WM**), MR differ in the fatty acid (**FA**) profile and triglyceride (**TG**) structure, which affects digestion dynamics and lipid metabolism (Babawale et al., 2018; Wilms et al., 2024a). Among vital lipids, cholesterol plays a critical role in maintaining membrane fluidity, regulating stress, and serving as a precursor for bile acids and steroid hormones (Arfuso et al., 2017; Babawale et al., 2018). Differences in cholesterol metabolism have been observed between breast-fed and formula-fed infants, with breast milk resulting in higher plasma cholesterol concentrations (Friedman and Goldberg, 1975; Woollett and Shah, 2023, Xu et al., 2013).

In calves, feeding an MR containing 14% (DM basis) with highly unsaturated fats, such as soybean and corn oils (∼55% of total FA as PUFA), resulted in higher plasma total cholesterol and low-density lipoprotein (**LDL**) cholesterol compared with an MR containing tallow (Wiggers et al., 1977; Richard et al., 1980). In recent studies, feeding MR ad libitum (30% fat) with a blend of rapeseed and coconut fats led to higher plasma total cholesterol in calves compared with MR containing a blend of lard and dairy cream (Wilms et al., 2024b). Unlike what has been reported in infants, this suggests that high dietary PUFA in MR may increase blood cholesterol in calves. Consistently, Leplaix-Charlat et al. (1996) reported higher total cholesterol concentration in calves fed a MR with soybean oil than tallow (∼23% fat, DM basis).

Blends of palm and coconut fats are one of the most common sources used for inclusion in MR for calves (Hu et al., 2023). As reported by Mellors et al. (2023), such fat blends have a high saturation level (62% SFA, % total FA) and low PUFA content (7%), which compares to bovine milk fat (71% SFA and 3% PUFA). In contrast, fat blends of rapeseed and coconut fats comprise approximately 52% SFA, 34% MUFA, and 13% PUFA (Wilms et al. 2024a). Consequently, feeding an MR with a blend of palm and coconut fats is unlikely to elevate blood cholesterol concentrations, given its high saturation level and low PUFA content. However, Echeverry-Munera et al. (2021) reported markedly higher plasma cholesterol in calves fed a high-fat MR (23% fat, DM basis) with palm and coconut fats compared with a low-fat MR (17% fat) containing the same fat blend. From this study, it was unclear whether the increase in plasma cholesterol was solely driven by higher fat intake or a combination of intake level and fat composition. Indeed, increased fat intake from MR containing lard and dairy cream did not lead to higher plasma cholesterol in calves fed ad libitum than in calves fed MR with a blend of rapeseed and coconut fats (Wilms et al. 2024b).

Thus, it was hypothesized that feeding an MR with a blend of palm and coconut fats or a blend of lard and dairy cream would not elevate plasma cholesterol concentrations, unlike feeding an MR with a blend of rapeseed and coconut fats. This study evaluated how fat composition in MR formulated with animal or vegetable fats affects plasma cholesterol in dairy calves.

This study is part of a larger experiment and partial data were published in 2 companion articles (Wilms et al., 2024a; Ghaffari et al., 2024). Forty-five Holstein-Friesian male calves were obtained from dairy farms located within a 30 km radius of the research facility. The enrollment period was between July 20 and October 26, 2020. The colostrum protocol was standardized, where calves received 3.0 to 4.0 L within the first 3 h after birth (minimum of 22% Brix), followed by 2 feedings of 2.0 L within 24 h after birth. Thereafter, calves were given 3.0 L of MR (Sprayfo Delta, Trouw Nutrition, the Netherlands) twice daily until the transportation day. Upon arrival at the research facility (2.3 ± 0.82 d of age; mean ± SD), calves were weighed, and serum IgG was measured between 36 to 72 h after birth using a portable Multi-Test Analyzer (DVM Rapid Test II, Vetlab), which required a minimum IgG of 10 g/L. Calves were housed indoors in individual pens (2.71 m^2^ per calf) bedded with flax straw. Calves were blocked according to their age and day of arrival. Within each block of 3 calves, animals were randomly assigned to 1 of 3 MR treatments (n = 15 calves per treatment): (1) a fat blend consisting of 80% of coconut and rapeseed oils (60% rapeseed oil, 20% fully hydrogenated rapeseed oil, and 20% coconut oil) added to 20% of refined coconut oil, which led to a final percentage of 65% rapeseed and 35% coconut fats (**RC**); (2) 65% palm and 35% coconut fats (**PC**); and (3) 65% packers lard and 35% liquid dairy cream (**LD**). The randomization was performed using the Microsoft Excel (Microsoft Corp.) random function [RANDBETWEEN (0,10000)]. Fat blends were formulated to resemble the FA profile of bovine milk fat using only 2 fat sources (Wilms et al., 2024a). While using only 2 fat sources, the RC and PC MR treatments represent 2 distinct approaches for formulating the fat composition of MR using only vegetable fats. The LD treatment represented an attempt to align with the FA profile of milk fat without including only bovine milk fat, as this would defeat one of the main purposes of MR, which is to increase the net dairy product yields of dairy farms. During MR production, the fats were spray-dried and subsequently encapsulated in a protein matrix. All MR were formulated to contain 63% skim milk powder, 30% crude oils and fats, 5% sweet whey powder, and 2% premix to include vitamins and minerals (Trouw Nutrition, Deventer, the Netherlands). This resulted in isoenergetic and isonitrogenous MR treatments with 36% lactose, 30% fat, 25% CP, and 6% ash (DM basis) with the intent to resemble the macronutrient profile of bovine WM. Calves were fed a MR allowance of 6.0 L/d from d 1 to 5, 7.0 L/d from d 6 to 9, and 8.0 L/d from d 10 to 35 in 2 equal meals fed at 0630 and 1730 h. Milk replacers were reconstituted with water using a milk shuttle (Urban MS100, Wüsting, Germany), at a concentration of 135 g/L (13.5% solids), and supplied in a nipple bucket at 40°C. Water and chopped wheat straw were offered ad libitum in plain buckets and refreshed daily. To isolate the effects of the MR treatments, no starter feed was fed.

Individual intakes of chopped straw, water, and MR were measured daily by weighing unconsumed amounts. Fecal consistency was visually evaluated by taking photographs of feces after the morning meal until d 21 after arrival. The decision to consider only the first 21 d was based on the period where morbidity and mortality from diarrhea in calves is the highest (Urie et al., 2018). The scoring system was based on Renaud et al. (2020), in which scores of 0 and 1 were considered as the absence of diarrhea (score 0 in this paper), 2 was considered mild diarrhea with the presence of wet feces (score 1 in this paper), and 3 was considered severe diarrhea with watery feces (score 2 in this paper). Calves were weighed at arrival and weekly thereafter until d 35 after arrival at 1300 h. At the same time points, blood samples were collected in a 9-mL tube with lithium heparin (BD Vacutainer, Becton Dickinson) from the jugular vein. All tubes were immediately placed on ice and centrifuged at 1,500 × *g* for 15 min at 4°C for 10 min (Rotina 380 R, Hettich, Tuttlingen, Germany). Plasma aliquots were stored in 1.5-mL cryotubes at −20°C. Samples were analyzed at Utrecht University (the Netherlands) as described by Wilms et al. (2024a). The analysis of macronutrients and minerals in the MR treatments is presented in Wilms et al. (2024a). The FA profile of the MR was analyzed by QLIP (Zutphen, the Netherlands; NEN-ISO 15885; Table 1).

**Table 1:** Fatty acid composition of experimental milk replacers

Fatty acid (% of total FA)	Treatment^1^		
	RC	PC	LD
C4:0	0	0.06	0.88
C6:0	0.27	0.15	0.63
C8:0	2.73	1.37	0.62
C10:0	2.08	1.16	1.11
C12:0	16.8	9.02	4.16
C14:0	6.62	4.86	5.17
C16:0	8.10	24.0	27.2
C17:0	0.05	0.28	0.36
C18:0	14.6	13.9	13.5
Total SFA^2^	52.0	54.9	53.9
Total *cis*-MUFA^3^	34.2	34.2	34.9
Total *trans*-MUFA	0	0.24	1.08
Total MUFA	34.2	34.5	36.0
C18:2 *cis*-9,12 (LA)^4^	9.62	8.50	7.30
C18:3 *cis*-9,12,15 (ALA)^5^	3.2	0.66	0.72
Total PUFA^6^	12.8	9.50	8.50
Total n-3 PUFA	3.20	0.73	0.79
Total n-6 PUFA	9.62	8.77	7.71
n-6/n-3 PUFA	3.01	12.0	9.76

1 Treatments (n = 15 calves/treatment): RC = 65% rapeseed and 35% coconut fats; PC = 65% palm and 35% coconut fats; LD = 65% packers lard and 35% dairy cream.

2 SFA = ∑C4:0 to C22:0.

3 MUFA = ∑MUFA: C14:1 to C20:1.

4 LA = linoleic acid.

5 ALA = α-linolenic acid.

6 PUFA = ∑PUFAn-6: LA; C20:3n-6; C20:4n-6; ∑PUFAn-3: C18:3n-3; C22:5n-3.

Continuous variables were analyzed using a mixed-effects model with PROC MIXED in SAS (9.4M6, SAS Institute Inc., Cary, NC). The calf was the experimental unit, and the statistical model used is as follows:

Y_ijklm_ = μ + T_i_ + δ_ij_ + P_k_ + (T × P)_ik_ + B_l_ + ε_ijklm_,

where Y_ijklm_ is the dependent variable; µ is the overall mean; T_i_ is the fixed effect of treatment; δ_ij_ is the random effect; P_k_ is the fixed effect of the week entering the model as a repeated measure; (T × P)_ik_ is the fixed effect interaction between the treatment and the week; B_l_ is the random effect of the block; and ε_ijklm_ is the random error. In the case of BW, arrival BW was used as the baseline covariate. The autoregressive covariance [AR(1)] structure was used as variables had equally spaced time points. Data that did not meet the assumptions of normality of residuals were tested following the Box-Cox power transformation, where the lambda (λ) generated corresponded to the recommended conversion of the data to achieve a normal distribution. A significant treatment effect was contrasted with the LSMEANS statement using the PDIFF option of the MIXED procedure in SAS. The results in the tables and figures are presented as the LSM with SEM. Fecal scores were analyzed using mixed-effects logistic regression using PROC GENMOD in SAS. Statistical significance was declared at *P* ≤ 0.05, and the threshold for trends was set at 0.05 < *P* ≤ 0.10.

The BW (45.6 ± 3.97 kg; mean ± SD; *P* = 0.99) and serum IgG concentration measured at arrival (20.4 ± 6.7 g/L; mean ± SD; *P* = 0.91) did not differ among treatments. Dietary treatments did not affect BW (*P* = 0.20), water intake (*P* = 0.42), or straw intake (*P* = 0.74). A treatment-by-time interaction was also detected for plasma total TG (*P* = 0.05), where calves fed PC had higher plasma TG than those fed LD on wk 2, whereas RC did not differ from other treatment groups (Figure 1A). There was a treatment-by-time interaction for plasma total cholesterol (*P* = 0.01) and HDL cholesterol (*P* = 0.03), where total (Figure 1B) and HDL cholesterol (Figure 1C) concentrations were higher in calves fed RC than in those fed LD between wk 2 and 5, and higher in calves fed RC than in those fed PC between wk 3 and 5. In addition, plasma total and HDL cholesterol concentrations were higher in calves fed PC than in those fed LD in wk 4 and 5. Additionally, a treatment-by-time interaction was also detected for plasma LDL cholesterol concentrations (*P* = 0.05; Figure 1D), which presented the same differences as plasma total cholesterol, except for wk 5. In wk 5, plasma LDL cholesterol was higher in calves fed RC than in those fed LD, whereas calves fed PC did not differ from the other groups. The proportion of HDL cholesterol (Figure 1E) and LDL cholesterol (Figure 1F) in total cholesterol did not differ. Calves fed RC had or tended to have less abnormal fecal scores than calves fed PC (*P* = 0.02) and LD (*P* = 0.06) in wk 2.

**Figure 1:**
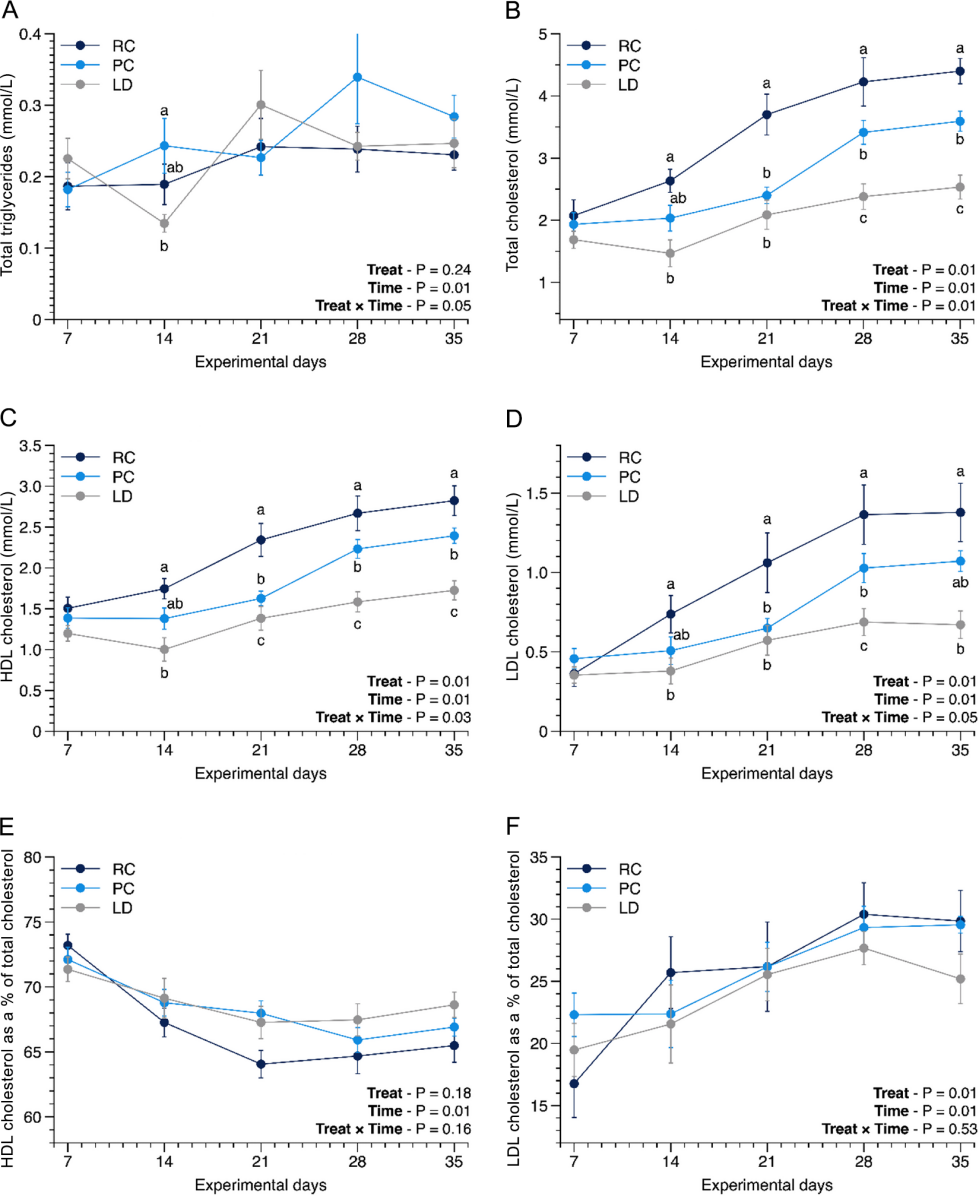
Total plasma triglyceride (A), total cholesterol (B), HDL cholesterol (C), LDL cholesterol (D), HDL cholesterol as a percentage of total cholesterol (E), and LDL cholesterol as a percentage of total cholesterol (F) according to the treatment. Treatments (Treat; n = 15 calves/treatment): RC = 65% rapeseed and 35% coconut fats; PC = 65% palm and 35% coconut fats; LD = 65% packers lard and 35% liquid dairy cream. Standard error bars were computed on raw data to better illustrate the observed differences in variability between treatments.

The elevated total plasma cholesterol observed in calves fed RC may be attributed to the higher PUFA content of the MR diet, based on earlier findings in which calves fed MR high in PUFA had increased plasma total cholesterol (Wiggers et al., 1977; Wilms et al., 2024b). Polyunsaturated FA are more evenly distributed among plasma lipid fractions in nonruminant species (Noble, 1979). This characteristic may explain the differences observed between infants and calves in circulating cholesterol when fed MR containing PUFA-rich oils. For example, in infants, most long-chain PUFA are transported primarily as phospholipids rather than as cholesteryl esters or TG (Prentice et al., 2015). Moreover, dietary PUFA appears to promote the redistribution of cholesterol between tissues and plasma in infants while maintaining a stable balance of total body cholesterol (Spritz et al., 1965). In addition to an increase in total cholesterol, Richard et al. (1980) reported a shift from HDL cholesterol to LDL cholesterol in calves fed MR containing corn and soybean oil over a 15-wk period, unlike calves fed an MR with tallow. In the current study, calves fed RC had a consistently higher proportion of LDL cholesterol than calves fed LD. This may be linked to changes in the excretion or synthesis of cholesterol in calves as an adaptation to high PUFA in MR (Barrows et al., 1980). Furthermore, the greater plasma HDL cholesterol of calves fed RC may be attributed to the activity of lecithin-cholesterol-acyltransferase (**LCAT**), which plays a crucial role in the synthesis of cholesteryl esters and the regulation of HDL and LDL cholesterol synthesis (Bauchart, 1993). Ruminant LCAT, in particular, exhibits high specificity for the transfer of PUFA, especially linoleic acid, which was more abundant in the RC formula (Noble et al., 1972).

In contrast to PUFA-rich oils, dietary palm and coconut fats have been shown to increase plasma cholesterol and LDL cholesterol in humans. However, when compared with butter, palm and coconut fats have been shown to lower LDL cholesterol (Boateng et al., 2016; Wallace, 2019; Rosqvist and Niinistö, 2024). Because of the high SFA and low PUFA content of palm fat, it was hypothesized that the plasma total cholesterol of calves fed PC would be similar to that of calves fed LD, but this was not confirmed by the results. This means that factors other than dietary PUFA in MR may affect plasma cholesterol. Palm fat is rich in palmitic acid (C16:0), which has been linked to elevated plasma cholesterol in humans (Kromhout et al., 1995; Rosqvist and Niinistö, 2024). Palmitic acid may suppress the inhibition of LDL receptors and enhance very low-density lipoprotein secretion from the liver, thereby increasing plasma LDL cholesterol (Spady et al., 1993). However, the proportion of C16:0 was lower in the RC MR (24%, % of total FA) than in the LD MR (27%) and, as observed in the results, calves fed RC had higher plasma LDL cholesterol than LD. This suggests that the high plasma cholesterol in calves fed PC was not related to the C16:0 content of the diet, but could rather be related to differences in TG structure. Although the C16:0 content is similar between palm and animal fats, less than 10% of C16:0 in palm fat is located at the sn-2 position on the TG backbone, compared with 20% to 30% in lard (Kritchevsky et al., 1998) and 30% to 45% in milk fat (Bracco, 1994; Zhao et al., 2005). Interesterification of dietary palm olein in human studies increased the percentage of C16:0 in sn-2 from 9% to 39%, which was preserved in chylomicrons postabsorption in healthy humans (Sanders et al., 2011). Thus, differences in TG structure can influence the amount of C16:0 reaching the liver, which may, in turn, influence cholesterol synthesis. Nevertheless, this remains to be studied in calves.

The reduction in abnormal fecal scores in calves fed RC in wk 2 after arrival may be related to the higher medium-chain fatty acid (**MCFA**) content of the RC diet. Indeed, MCFA have a higher digestibility than FA with longer chain length (Wallace, 2019), mainly because the pancreatic and lingual lipase show higher activity for FA with shorter chain length (C2:0–C8:0; Benito-Gallo et al., 2015). This may favor a reduction in fat metabolite retention time in the lumen of the intestine, decreasing organic accumulation throughout the intestinal tract and, thus, enhancing fecal consistency. In addition, MCFA exhibit antimicrobial properties (Hristov et al., 2004) that may act against enteric pathogens.

Including palm and rapeseed fats in MR resulted in higher plasma total cholesterol in calves, unlike a blend of lard and dairy cream. Although it is not possible to isolate the effects of individual FA, it could be that imbalances in PUFA and large differences in TG structure between vegetable and animal fats may affect cholesterol metabolism in calves.
